# Physician Orders for Waiting Room Patients: Ethical Considerations

**DOI:** 10.5811/westjem.33481

**Published:** 2025-07-12

**Authors:** Nicholas Kluesner, Jennifer Chapman, Monisha Dilip, James H. Paxton, Karen Jubanyik, Paul Bissmeyer

**Affiliations:** * Iowa Methodist Medical Center, Department of Emergency Medicine, Des Moines, Iowa; † HCA Florida Orange Park Hospital, Department of Emergency Medicine, Orange Park, Florida; ‡ Yale University School of Medicine, Department of Emergency Medicine, New Haven, Connecticut; § Wayne State University School of Medicine, Department of Emergency Medicine, Detroit, Michigan; ** Orange Park Hospital, Department of Emergency Medicine, Jacksonville, Florida

## Abstract

With increasing emergency department (ED) boarding and crowding, EDs have introduced several novel care-delivery initiatives including split-flow models (e.g., fast tracks), non-linear patient flow models (e.g., protocol bays), nursing triage order sets, physician-in-triage, and the use of non-traditional care areas (e.g., ED hallways). One such emerging practice is the placement of orders for patients in the waiting room (WR) by physicians prior to in-person physician evaluation (e.g., based on triage documentation and the patient’s medical record). This paper describes key ethical obligations to WR patients that support this practice, as well as other considerations that must be balanced against these obligations, including potential risks.

## INTRODUCTION

The Emergency Medical Treatment and Labor Act (EMTALA), a federal law enacted in 1986, underscores the obligation of emergency departments (ED) in the United States to provide care for all people, including those who are ultimately found to have a low-acuity condition.^1^ Under this federal mandate, with mounting pressure of ED crowding and boarding, many hospitals have experimented with novel care delivery initiatives. It is important to note that none of these novel delivery-care initiatives absolve the foundational EMTALA obligation for a medical screening exam by qualified medical personnel. These processes include split-flow models (e.g., fast tracks), non-linear patient flow models (e.g., protocol bays), nursing triage order sets, physician-in-triage (PIT), and the use of non-traditional care areas (e.g., ED hallways).

Studies evaluating the effects on operational metrics of these initiatives, which largely look at PIT, are weak and show mixed results. While some studies have reported decreased time in room (TIR)^2^ or ED length of stay (LOS)^3^–^10^ associated with this practice, others have found no significant reduction in LOS.^11^,^12^ Among these attempts to improve the care and efficiency of crowded EDs is the practice of physicians placing orders on patients in the waiting room (WR)—beyond standardized nursing triage protocols—based upon the written triage report but prior to face-to-face evaluation of the patient by a physician. There is no empiric data published regarding the efficacy of this practice, or its impact on patient quality outcomes or resource utilization. While this remains important and needed research, objections to this practice often cite philosophical rather than empiric concerns.

In this paper, we explored the ethical implications of this practice of placing orders on waiting room patients because each novel care-delivery initiative deserves its own unique analysis of benefits and risks. This practice differs from other types of accelerated ordering models, such as the use of routine triage nursing protocols or a PIT model, as it lacks an in-person evaluation by the emergency physician (EP) but requires their active engagement in WR patient care. In our exploration of this topic, we first define ethical obligations held for all ED patients including those unique ethical obligations to WR patients who have not yet received in-person evaluation by an EP. These considerations support placing orders to the potential benefit of WR patients. We then explore the risks and logistical issues to this practice that must be considered. Our goal in this ethical analysis iss to provide focused guidance for clinicians and administrators to appropriately use this evolving practice in the crowded ED.

## OBLIGATIONS TO ALL PATIENTS

Emergency physicians are bound by both a moral and legal obligation to evaluate and treat all patients seeking emergency care,^13^–^15^ for it is only because of their ED care that an emergent medical condition may be ruled out or managed. To ensure the greatest amount of good is achieved for the greatest number of patients, those patients with the highest acuity of illness are generally prioritized over those presenting with a lower acuity condition.^16^ This is the moral and pragmatic foundation for triage. Triage-driven diagnostic testing such as electrocardiograms (ECG), labs, and imaging performed in the WR may aid in more accurately stratifying patient acuity.^17^

Considering this obligation to all patients—both low and high acuity, roomed and waiting—it is important to emphasize that the strongest obligation of clinical ED staff is to provide care for the highest acuity patients.^2^ As more attention is turned to triage and WR medicine with ED crowding, recognition that WR patients are more likely to leave without being seen (LWBS) is inevitable.^6^ The ED processes should optimize triage accuracy and efficiency, improve overall access to care, and reflect patient-oriented outcomes, yet these processes cannot compromise the care of higher acuity patients.^4^ This is in keeping with EDs obligations to all patients.

## OBLIGATIONS TO WAITING ROOM PATIENTS

For the patient presenting to the ED requesting evaluation, we have already established the clear obligation EPs have to the WR patient. Because the EP and WR patient have not initiated a traditional physician-patient relationship prior to in-person assessment, however, this may be contested or diminished. Nonetheless, the EP’s actions and priorities are clearly and undeniably linked to the WR patient’s well-being and, thus, require dedicated attention here.

Emergency physicians are not able to uncouple the effects of their actions on WR patients. As an extreme example, it would be clearly ethically unacceptable for an EP who recognizes a potential emergent situation (e.g., stroke alert) to leave for a coffee break. Similarly, pursuing non-emergent care of patients while they occupy an ED bed, without weighing the negative effects of further delays in care for WR patients, fails to respect the principle of non-maleficence. In other words, an ethical obligation and link between the EP and the WR patient exists, even prior to the establishment of a direct patient-physician relationship.

Conversely, actively engaging in the care of WR patients before in-person physician evaluation can provide expedited care and enhanced triage prioritization for waiting patients. For example, a concerning ECG obtained on an elderly WR patient with generalized weakness will likely result in earlier recognition of critical hyperkalemia and improved outcomes. This simple example illustrates how the placement of orders on WR patients can clearly benefit patients; thus, the justification for placing such orders can be said to be founded upon the ethical principle of beneficence toward WR patients.

Commensurate with these obligations to benefit and avoid harm of WR patients by EP action or inaction are two responsibilities for the EP:

1. The EP must have a good understanding of the resources available to them as well as the need for prioritization among patients needing those resources. This is triage, fundamentally. The stratification of patient acuity can and should be enhanced by appropriate testing orders to accomplish the utilitarian goal of triage.
2. EPs have a responsibility to optimize each WR patient’s care, despite the limitations of triage and regardless of whether they have not yet personally evaluated the patient. Placing orders on WR patients is one way this can be accomplished, which may benefit those patients in the form of enhanced triage information, enhanced flow through the ED, and screening for acute conditions not immediately obvious from triage nursing assessments.

## LIMITATIONS OF THE OBLIGATION TO PROVIDE CARE

While we defended an ethical obligation to WR patients that could necessitate EP orders, there must also be a prima facie limitation on these obligations as there is an important, added value to a patient’s evaluation by a physician. An EP’s history and examination are the gold standard for the evaluation of an emergent medical condition because of their ability to elicit critical findings and weigh clinical significance. Furthermore, the brief and potentially less-private nature of triage may lead to incomplete or withheld information, limiting the EP’s ability to make appropriate decisions prior to an in-person evaluation. We also acknowledge, as highlighted earlier, that these arguments are conceptual and philosophical in nature and that there is no empiric data on patient-oriented outcomes of EP orders on patients in the WR to validate any potential benefit; however, there is equally no empiric data of any potential harms. Future research in this area would be a valuable next step given that the ethical framework here supports exploring this practice.

The practical execution of orders on WR patients will always be limited by the ED staff’s bandwidth to accomplish these tasks, which is likely already spread thin by a crowded ED precipitating the conditions to consider this practice. In keeping with appropriate triage and the obligations to all patients, it would be inappropriate for ED staff to prioritize WR orders over orders for patients with higher triage priorities who are already under physician evaluation and management. A physician ordering tests on WR patients must consider these logistical considerations and limitations.

## RISKS OF WAITING ROOM ORDERS AND OTHER CONSIDERATIONS

### Stewardship and Resource Utilization

In emergency medicine, stewardship is not just a fiscal concern but an ethical imperative. The American College of Emergency Physicians advocates for resource allocation that maximizes patient benefit while minimizing unnecessary expenditure as an ethical obligation to stewardship.^18^ Hence, when EPs order tests based solely upon triage records, the potential to over-use resources poses an ethical risk. This is a risk that must be weighed against potential benefits to the patient previously established.

The decision to exclude specific tests in the WR setting such as magnetic resonance imaging or computed tomography (CT) is nuanced and the cost-benefit analysis complex; blanket exclusions may not always serve patient interests. Instead, a case-by-case approach, as informed by national guidelines and EP judgment, is recommended.^19^ While these modalities may have higher risks and limitations, they may also have greater benefits for select patient populations.

### Over-testing Consequences for the Patient

Over-testing can lead to anxiety and subsequent unnecessary testing in the case of false positives. While most repeated tests would not be medically dangerous to the patient, some (e.g., biopsies) could pose an additive risk. Additionally, ordering CT prior to examination, for example, could bring harm in the form of unnecessary costs and radiation exposure.^20^

### Patient Communication and Consent

The *New York Times* and other media outlets have highlighted the fragile nature of patient trust in emergency settings.^21^ Such trust is a paramount consideration when patients agree to undergo tests before seeing a physician. To honor this trust, the ED must strive for a transparent strategy to communicate the nature and necessity of the WR orders. This may include notices in waiting areas and, perhaps more importantly, verbal communication from medical staff, which can address any patient concerns and ensure that genuinely informed consent has been obtained. Ultimately, the onus is on healthcare professionals to ensure that consent goes beyond a mere signature to an authentic understanding.^22^

Shared decision-making on the choice of diagnostic test is also important. Shared decision-making maximizes autonomy, is associated with physician comfort with ordering fewer tests,^23^ and should be the preferred or default approach in most ED situations.^24^ Shared decision-making may not be available when orders are placed on WR patients, but patients should be made aware that they have the autonomy to decline testing prior to physician evaluation.

### Follow-up on Results

Follow-up procedures for patients who leave without being seen (LWBS) can be fraught with ethical and operational challenges. Direct patient access to test results mandated by the 21^st^ Century Cures Act, enacted in 2016,^25^ may appear to empower patients but could also contribute to patients making misinformed decisions about their need for further medical care.^26^ Patients must be cautioned early in the triage process that any test results obtained from screening tests ordered in the WR should not be interpreted by the patient alone.

The ED’s role as the primary safety net for many vulnerable populations adds a layer of ethical responsibility for ensuring appropriate follow-up on especially critical medical findings after the ED encounter. The follow-up implications for results after a patient LWBS should be part of the informed consent process.

### Liability Implications

The potential for malpractice liability is a significant concern for EPs who order tests for patients whom they have not personally evaluated. The central question of whether such preemptive orders establish a physician-patient relationship carries substantial legal implications. If a relationship exists, the physician could be held to the same standards of care as when they have conducted a complete evaluation. Comparing this to the indirect medical control model used in emergency medical services (EMS) might provide a suitable framework for understanding the legal differences involved. Under this model, EMS personnel operate under the oversight of physicians who are granted added legal protections due to the indirect nature of their patient care.^27^

However, the applicability of such protections to the ED setting is still a matter of legal interpretation. Despite the EP’s physical proximity to patients in the WR, the indirect and asynchronous orders on WR patients are more akin to EMS oversight than the traditional medical care model, which we believe should confer the added legal protections. This distinction is crucial in determining the appropriate threshold for liability, which hinges upon a standard of recklessness rather than negligence.^28^

### The Slippery Slope of Systemic Acclimatization

The routine use of WR orders, as a stopgap measure in the setting of significant ED crowding, could lead to acclimatization to systemic issues such as crowding and resource limitations. If such practices become normalized, there is a risk of diminishing the standards of emergency care. Ethical analyses warn of the potential long-term implications of accepting suboptimal conditions as standard.^29^ Even if certain throughput metrics (e.g., LWBS) show improvement, these benefits should be scrutinized for their patient-oriented value and must be weighed against the long-term potential for such practices to establish a lower standard of care for ED patients. By accommodating such practices, there may be less impetus to address the underlying causes of crowding and find sustainable, systemic solutions.

## CONCLUSION

Data is sparse regarding the efficiency and clinical impact of physician orders for patients in the waiting room prior to in-person evaluation. Therefore, the decision to engage in this practice should be reliant upon the EP’s clinical judgment, driven by an obligation to reserve its use for situations in which the benefits of this approach outweigh the risks. The benefits—potentially enhancing patient triage and advancing their medical care—are material and should not be dismissed wholesale despite the potential risks and limitations of this practice. However, the limitations and risks of this practice must also be acknowledged and mitigated, including challenges with overutilization, informed consent, and acclimatization. These benefits and risks may be especially important in the setting of extreme ED crowding, where this practice is most likely to be necessary.

## References

[1] (2023). Emergency Medical Treatment & Labor Act (EMTALA).

[2] Stauber MA (2013). Advanced nursing interventions and length of stay in the emergency department. J Emerg Nurs.

[3] Russ S, Jones I, Aronsky D (2010). Placing physician orders at triage: the effect on length of stay. Ann Emerg Med.

[4] Rowe BH, Villa-Roel C, Guo X (2011). The role of triage nurse ordering on mitigating overcrowding in emergency departments: a systematic review. Acad Emerg Med.

[5] White BA, Brown DF, Sinclair J (2012). Supplemented Triage and Rapid Treatment (START) improves performance measures in the emergency department. J Emerg Med.

[6] Rogg JG, White BA, Biddinger PD (2013). A long-term analysis of physician triage screening in the emergency department. Acad Emerg Med.

[7] Sokoloff C, Daoust R, Paquet J (2014). Is adequate pain relief and time to analgesia associated with emergency department length of stay? A retrospective study. BMJ Open.

[8] Marshall JR, Katzer R, Lotfipour S (2017). Use of physician-in-triage model in the management of abdominal pain in an emergency department observation unit. West J Emerg Med.

[9] Li Y, Lu Q, Du H (2018). The impact of triage nurse-ordered diagnostic studies on pediatric emergency department length of stay. Indian J Pediatr.

[10] Hughes JA, Brown NJ, Chiu J (2020). The relationship between time to analgesic administration and emergency department length of stay: a retrospective review. J Adv Nurs.

[11] Traub SJ, Bartley AC, Smith VD (2016). Physician in triage versus rotational patient assignment. J Emerg Med.

[12] Begaz T, Elashoff D, Grogan TR (2017). Differences in test ordering between nurse practitioners and attending emergency physicians when acting as provider in triage. Am J Emerg Med.

[13] Knowles K, Beltran G, Grover L (2020). Emergency department operations I: Emergency medical services and patient arrival. Emerg Med Clin North Am.

[14] Innes K, Jackson D, Plummer V (2015). Care of patients in emergency department waiting rooms – an integrative review. J Adv Nurs.

[15] Innes G, Pauls M, Campbell S (2019). CJEM Debate Series: #HallwayMedicine – Our responsibility to assess patients is not limited to those in beds; emergency physicians must assess patients in the hallway and the waiting room when traditional bed spaces are unavailable. Can J Emerg Med.

[16] Moskop JC, Geiderman JM, Marshall KD (2019). Another look at the persistent moral problem of emergency department crowding. Ann Emerg Med.

[17] Scheuermeyer F, Christenson J, Innes G (2010). Safety of assessment of patients with potential ischemic chest pain in an emergency department waiting room: a prospective comparative cohort study. Ann Emerg Med.

[18] A Ethics Committee (2002). Resource Utilization in the Emergency Department: The Duty of Stewardship.

[19] American College of Radiology (2023). ACR Appropriateness Criteria®.

[20] Müskens JLJM, Kool RB, van Dulmen SA (2022). Overuse of diagnostic testing in healthcare: a systematic review. BMJ Qual Saf.

[21] Brody JE (2009). Well-chosen words in the doctor’s office.

[22] Schmidt TA, Salo D, Hughes JA (2004). Confronting the ethical challenges to informed consent in emergency medicine research. Acad Emerg Med.

[23] Kanzaria HK, Hoffman JR, Probst MA (2015). Emergency physician perceptions of medically unnecessary advanced diagnostic imaging. Acad Emerg Med.

[24] Probst MA, Kanzaria HK, Schoenfeld EM (2017). Shared decision making in the emergency department: a guiding framework for clinicians. Ann Emerg Med.

[25] United States Congress (2016). 21st Century Cures Act.

[26] Petrovskaya O, Karpman A, Schilling J (2023). Patient and health care provider perspectives on patient access to test results via web portals: scoping review. J Med Internet Res.

[27] Baker J, Cole J, StatPearls (2023). EMS medical oversight of systems. StatPearls.

[28] Chapman MK (2020). The difference between negligence and malpractice.

[29] Nair T, Savulescu J, Everett J (2017). Settling for second best: when should doctors agree to parental demands for suboptimal medical treatment?. J Med Ethics.

